# Brain-Predicted Age Difference Moderates the Association Between Muscle Strength and Mobility

**DOI:** 10.3389/fnagi.2022.808022

**Published:** 2022-01-31

**Authors:** Brooke A. Vaughan, Janet E. Simon, Dustin R. Grooms, Leatha A. Clark, Nathan P. Wages, Brian C. Clark

**Affiliations:** ^1^Ohio Musculoskeletal and Neurological Institute (OMNI), Ohio University, Athens, OH, United States; ^2^School of Rehabilitation and Communication Sciences, Ohio University, Athens, OH, United States; ^3^School of Applied Health Sciences and Wellness, Ohio University, Athens, OH, United States; ^4^Department of Biomedical Sciences, Ohio University, Athens, OH, United States; ^5^Department of Family Medicine, Ohio University, Athens, OH, United States

**Keywords:** weakness, physical function, sarcopenia, brain aging, dynapenia

## Abstract

**Background:**

Approximately 35% of individuals over age 70 report difficulty with mobility. Muscle weakness has been demonstrated to be one contributor to mobility limitations in older adults. The purpose of this study was to examine the moderating effect of brain-predicted age difference (an index of biological brain age/health derived from structural neuroimaging) on the relationship between leg strength and mobility.

**Methods:**

In community dwelling older adults (*N* = 57, 74.7 ± 6.93 years; 68% women), we assessed the relationship between isokinetic leg extensor strength and a composite measure of mobility [mobility battery assessment (MBA)] using partial Pearson correlations and multifactorial regression modeling. Brain predicted age (BPA) was calculated from T1 MR-images using a validated machine learning Gaussian Process regression model to explore the moderating effect of BPA difference (BPAD; BPA minus chronological age).

**Results:**

Leg strength was significantly correlated with BPAD (*r* = −0.317, *p* < 0.05) and MBA score (*r* = 0.541, *p* < 0.001). Chronological age, sex, leg strength, and BPAD explained 63% of the variance in MBA performance (*p* < 0.001). BPAD was a significant moderator of the relationship between strength and MBA, accounting for 7.0% of MBA score variance [△*R*^2^ = 0.044, *F*(1,51) = 6.83, *p* = 0.01]. Conditional moderation effects of BPAD indicate strength was a stronger predictor of mobility in those with a great BPAD.

**Conclusion:**

The relationship between strength and mobility appears to be influenced by brain aging, with strength serving as a possible compensation for decline in neural integrity.

## Introduction

The population of individuals over age 65 in the United States is expected to nearly double from 43.1 to 83.7 million by the year 2050 (Ortman et al., 2014). This shifting age demographic carries significant health, economic, and social implications and highlights the need to develop preventative and restorative interventions to facilitate healthy aging. Unfortunately, decline in functional mobility [i.e., a person’s ability to move independently and safely in a variety of environmental contexts to accomplish functional tasks (Bouça-Machado et al., 2018)] is a common consequence of aging, with as many as 35% of individuals over 70 and most individuals over 85 reporting difficulty with ambulation or activities of daily living (Cummings et al., 2014; Musich et al., 2018). Functional mobility deficits have been linked to increased fall risk, poorer psychosocial health, and greater health expenditure (Musich et al., 2018). In addition, mobility limitations are predictive of disability and mortality (Newman et al., 2006b).

The determinants of mobility are multi-factorial. Muscle weakness is one factor repeatedly shown to be associated with reduced functional mobility and future functional declines and mortality in older individuals (Visser et al., 2000; Newman et al., 2006a; Manini et al., 2007). Scientists and clinicians have long posited that age-related loss of lean mass is the primary mediator between weakness and mobility impairment in older adults. However, emerging evidence has highlighted the importance of central neural processes in muscle strength capacity (Clark et al., 2014; Carson, 2018; Clark and Carson, 2021). Age-related decreases in overall brain volume (Storsve et al., 2014), cortical thinning (Thambisetty et al., 2010; Storsve et al., 2014), and microvascular irregularities (Maniega et al., 2015) have been linked to frailty (Lu et al., 2020) and impaired functional mobility (de Laat et al., 2012; Pinter et al., 2018; Lockhart et al., 2021). Thus, we postulate that indices of brain pathology and aging may serve to moderate the well-known association between muscle strength/weakness and mobility in older adults.

**FIGURE 1:**
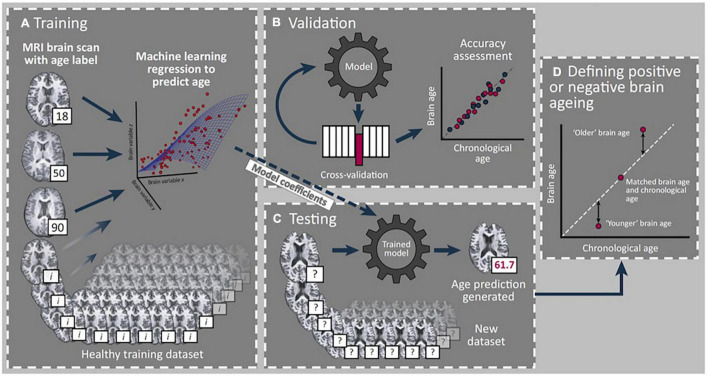
Summary of brain age prediction using a supervised machine learning process. **(A)** Structural T-1 MRI scans labeled with chronological age from a training set of healthy individuals are loaded into a machine learning regression model. **(B)** Validation of model accuracy is conducted using cross-validation methods from a portion of the original dataset excluded from the model. Model generated predicted age values are compared with actual age values to determine model accuracy. **(C)** Model coefficients from the trained model are applied to a new test dataset to determine individual brain age prediction (61.7 years in this example). **(D)** A standardized metric for statistical comparison is created (brain-predicted age difference) by subtracting chronological age from predicted age to reflect rate of brain aging, with positive and negative values indicating older and younger brains, respectively. *Reprint permission from Elsevier from Trends in Neuroscience, 40 (12), Cole J. H. and Franke K., Predicting age using neuroimaging: innovative brain ageing biomarkers, 681–90, 2017.

Magnetic resonance imaging (MRI)-derived estimates have recently garnered attention as one approach to reliably quantify brain age (Franke et al., 2010; Cole and Franke, 2017; Cole et al., 2017, 2019). Here, “brain-predicted age” (BPA) is derived from T1-weighted neuroimages using machine learning approaches that have previously been employed to quantify the relationship between structural MRI data and chronological age (Figure 1). Subtracting chronological age from the estimated BPA results in a BPA difference (BPAD) score, which effectively quantifies how an individual’s brain health differs from what would be expected for their chronological age (Franke et al., 2010; Cole and Franke, 2017; Cole et al., 2017). To date, researchers have linked accelerated brain aging to various pathological conditions including Alzheimer’s disease (Franke et al., 2010), diabetes (Franke et al., 2013), and obesity (Ronan et al., 2016). Longitudinal studies have even demonstrated that individuals with older BPAD display early signs of cognitive decline from childhood to midlife (Elliott et al., 2019) and are more likely to receive a subsequent dementia diagnosis (Biondo et al., 2021). Thus, BPAD has been proposed as a biomarker of age-related deterioration of the brain (Cole and Franke, 2017; Cole et al., 2017).

Despite the promising application of neuroimaging to estimate biological brain age to disease populations, only one study has investigated the influence of biological brain age on physical function measures in a community-dwelling individuals. Here, Cole et al. (2018) reported an association between positive BPAD (i.e., an “older” brain relative to ones years) and decreased walking speed, poorer lung function, and weaker grip strength in a large, longitudinal study cohort. These findings offer support to the evolving perspective of the role of brain aging in relation to mobility. However, no study has investigated the influence of BPAD as a moderator of the well-established relationship between muscle strength and mobility. Accordingly, the primary purpose of this study was to examine the relationship between isokinetic leg extensor strength and mobility with BPAD as a potential moderator of this relationship in a community-dwelling older population.

## Materials and Methods

### Overview of Study Design

Data presented in this report are derived from a larger study/dataset (UNCODE Study; NCT02505529). To be included in the original study, participants had to be ≥60 years of age, living independently and free from overt musculoskeletal and neurologic disease (see Table 1 for a complete description of UNCODE inclusion and exclusion criteria). The Ohio University Institutional Review Board approved this study, and all participants provided written informed consent in accordance with the Declaration of Helsinki.

**TABLE 1:** UNCODE inclusion and exclusion criteria.

INCLUSION
Age 60+ years (older adults) with no significant health issues or conditions that, in the investigator’s opinion, would limit the subject’s ability to complete the study per protocol or that would impact the capability to get an accurate measurement of study endpoints.
Body mass index between 18 and 40 kg/m^2^.
Willingness to undergo all testing procedures.
Able to read, understand, and complete study-related questionnaires.
Able to read and understand, and willing to sign the informed consent form (ICF).
**EXCLUSION**
Failure to provide informed consent.
Known neuromuscular or neurological conditions affecting somatosensory or motor function or control (e.g., hemiplegia, multiple sclerosis, peripheral neuropathy, Parkinson’s disease, Myasthenia Gravis, Ataxia, Apraxia, mitochondrial myopathy, etc.).
Unable to communicate because of severe hearing loss or speech disorder.
Severe visual impairment, which would preclude completion of the assessments.
Cancer requiring treatment currently or in the past 2 years (except primary non-melanoma skin cancer or *in situ* cervical cancer).
Any ADL disability.
Recent unexplained weight loss (>10 pounds in past month).
Hospitalization (medical confinement for 24 h), or immobilization, or major surgical procedure requiring general anesthesia within 12 weeks prior to screening, or any planned surgical procedures during the study period.
Chronic or relapsing/remitting gastrointestinal disorders such as inflammatory bowel disease and irritable bowel syndrome.
Known history of human immunodeficiency virus (HIV) antibody at screening.
Use of systemic glucocorticoids.
Severe pulmonary disease, requiring either steroid pills or injections or the use of supplemental oxygen.
Severe cardiac disease, including NYHA Class III or IV congestive heart failure, clinically significant aortic stenosis, recent history of cardiac arrest (within 6-months), use of a cardiac defibrillator, or uncontrolled angina.
Renal failure on hemodialysis.
Psychiatric conditions that warrant acute or chronic therapeutic intervention (e.g., major depressive disorder, bipolar disorder, panic disorder, schizophrenia) that in the investigator’s opinion interfered with the conduct of study procedures.
Unable to undergo Magnetic Resonance Imaging (MRI), Transcranial Magnetic Stimulation (TMS), or DEXA (e. g. body containing any metallic medical devices or equipment, including heart pacemakers, metal prostheses, implants or surgical clips, any prior injury from shrapnel or grinding metal, exposure to metallic dusts, metallic shavings or having tattoos containing metallic dyes, body dimensions exceeding capacity of MRI or DEXA). Note: This manuscript is an analysis from a larger study and the MRI and brain stimulation exclusion criteria, which are not presented here, were part of this larger study.
Unable to reliably undergo exercise or strength tests described for this study.
Participation in any clinical trial within 12 weeks prior to screening.
Limb amputation (except for toes) and/or any fracture within 24 weeks of study screening.
Conditions (such as myasthenia gravis, myositis, muscular dystrophy, or myopathy, including drug-induced myopathy) leading to muscle loss, muscle weakness, muscle cramps, or myalgia.
Acute viral or bacterial upper or lower respiratory infection at screening.
Abnormal or uncontrolled blood pressure at the screening visit defined as BP > 170/100 mmHg. If taking anti-hypertensive medication, had to have been on stable doses of medication for more than 3 months.

To characterize the study participants, we measured body composition (including estimates of appendicular and thigh lean mass) using DEXA (Hangartner et al., 2013; Tavoian et al., 2019) and moderate-to-vigorous intensity physical activity *via* accelerometry (Corbett et al., 2016). We quantified isokinetic (60°/s) leg extension strength and determined biological brain age estimates using T1 structural images and a previously validated machine-learning model (Cole et al., 2017). To capture the multi-dimensional nature of mobility, we measured functional performance using the mobility battery assessment (MBA) score, a multi-component evaluation of mobility that has been shown to be a more robust measure of self-reported lower extremity function and a more sensitive discriminator of mobility capacity in older individuals than single functional assessments (Riwniak et al., 2020). The specific methodological details related to our primary variables of interest have been outlined in previously published work (Clark et al., 2019, 2021; Wages et al., 2020), but a brief description is provided below.

### Muscle Strength

Isokinetic leg extension strength was assessed using a Biodex System 4 Dynamometer (Biodex Medical Systems Inc., Shirley, NY, United States). Specific operational procedures follow those used in the Health ABC study (Manini et al., 2007). In brief, subjects performed six isokinetic trials measured at 60°/s with a 30 s rest period between each trial. Average isokinetic strength of the highest three trials was calculated using the peak isokinetic torque output from 90° to 30° of leg flexion. Isokinetic strength was normalized to body weight for all subsequent analyses as this measure has been shown to be predictive of the subsequent development of severe mobility limitations (Manini et al., 2007).

### Brain Predicted Age

T1 structural images were acquired on a 3.0 T Philips Achieva scanner with a 16-channel head coil with whole-brain axial gradient-echo MPRAGE 3-D T1-weighted images [*TE*/*TR* = 3.4/7.4 ms, flip angle = 8, slice thickness = 1 mm (contiguous slices), Field of View (FOV) = 250 × 250 × 200 mm,1 mm^3^ resolution]. Participants were instructed to close their eyes during the scans. Scanner precision was assessed using repeat scans from a separate cohort of healthy, young adults who were scanned on two separate occasions (*n* = 7; age 25.43 ± 6.78 years).

Brain-predicted age was calculated using a previously validated Gaussian Process regression model published in other work (Cole et al., 2017). In brief, the brainageR model was trained on 3,377 healthy individuals (mean age = 40.6 years, *SD* = 21.4, range 18–92) from several large publicly available datasets. Model testing was subsequently performed on independent data representing 611 subjects, demonstrating a correlation with chronological age of 0.947 and a mean absolute error of 4.90 years (Cole et al., 2017). With respect to our data, we followed the procedure and employed the brainageR algorithm as previously established by Cole et al., 2015, 2017, 2019.^1^ Raw T1-weighted MRI scans for each participant underwent segmentation and normalization procedures using SPM12 and a customized version of FSL *slicesdir* was used to create PNG and index.html files for quality control. Normalized image files were then loaded into R using the RNfiti package to separate and mask gray and white matter and cerebrospinal fluid. To calculate BPA for each participant in the current dataset, a rotation matrix with 435 components from the brainageR model was applied using kernlab (Figure 1). BPA was then converted to the variable of interest, BPAD, by subtracting participants’ chronological age from their brain-predicted age estimate with positive and negative BPAD reflecting older and younger biological brains, respectively.

### Mobility Battery Assessment

The MBA includes both locomotor (6-min walk gait speed, time to complete the four-square step test and stair climb power) and non-locomotor (5× chair rise time and time to complete a complex functional task) functional measures. The overall MBA score is calculated using principal component analysis and reflects performance on all five tasks. MBA scores have a distribution of a mean of 0 and a standard deviation of 1.0 (Riwniak et al., 2020). Below we describe the testing involved in the individual components of the MBA score.

#### Six-Minute Walk Test

Participants were asked to walk as far as possible in 6 min by repeating a 60-m course that included a 30-m straight walkway and two 180° turns to the left. Participants were informed of the time remaining after each 60m lap and were provided with verbal encouragement. Gait speed for each participant was calculated by dividing the distance traveled during the task by 360 s.

#### Four Square Step Test

Participants were instructed to step over four pieces of tape placed on the floor in a plus sign (i.e., +) configuration in a predetermined sequence as quickly as possible. Trials were not considered successful if the participant touched the tape, did not follow the instructed stepping sequence or was unable to maintain balance. Time to complete the task was measured with a stopwatch to the nearest 0.01 s and performance scores reflect the mean of three successful trials.

#### Stair Climb Power

Participants were instructed to ascend a flight of eight stairs (∼18 cm rise) as quickly as possible without the use of upper extremity handrail support unless needed for safety. Time to complete the stair climb was measured using switch mats (Lafayette Instruments Model 54,060) interfaced with a digital timer to the nearest 0.01 s. Participants performed the task twice and results were averaged from both trials. Stair climb power was then calculated as: Power = [(body weight in kg) × (9.8 m/s^2^) × (stair height in meters)]/time in seconds where stair height was the sum height of all eight stairs.

#### 5× Chair Rise

From an erect sitting position, participants were instructed to fold their arms across their chest and stand and sit from a chair (∼46 cm high) five times consecutively. The time to complete the task was measured using a stopwatch to the nearest 0.01 s and participants performed only one trial.

#### Complex Functional Task

For this assessment, participants were asked to complete a composite skill previously described in other work (Wages et al., 2020). Briefly, subjects began the task seated on the floor and were asked to stand, lift a 4.5 kg weighted laundry basket, walk 1.5 m, and place the basket on a surface 0.75 m high. Time to complete the task was measured using a stopwatch to the nearest 0.01 s for two trials, with scores representing the mean of both trials. If a participant was unable to complete the task, or it took >30 s, a value of 30 s was assigned to that participant. If a participant was only able to complete the task once, the time on that trial represented their performance.

### Statistical Analysis

All statistical analyses were conducted using Statistical Package for Social Sciences (SPSS) version 25 (SPSS Inc., Chicago, IL, United States) with a significance level of 5% (two-tailed). All data was expressed as the mean ± SD for the descriptive statistics.

The first level analysis was to explore the relationship between chronological age, lower extremity strength, BPAD, and the overall MBA score and components of the MBA using bivariate Pearson correlation tests. Partial Pearson correlation coefficients were also used to examine the relationship between average isokinetic lower extremity extensor strength normalized to body weight, the MBA, and individual functional performance measures (i.e., 5× chair rise, etc.) with sex and chronological age included as covariates. In a separate analysis, BPAD was included as an additional covariate to reflect the hypothesized neural contributions to mobility in older individuals. Since the brainageR model does not automatically correct brain age estimates for the statistical influence of chronological age, both chronological age and BPAD were included in this analysis to capture the differential impact of biological brain and chronological age discrepancies across the lifespan (Le et al., 2018). Finally, partial Pearson correlation coefficients were used to investigate any potential relationship between BPAD and functional performance measures with sex and chronological age as covariates. The same analyses were also performed using lower extremity extensor strength normalized to body weight as an additional covariate.

In a second level of analysis, a hierarchical multiple regression was conducted to investigate the predictive value of lower extremity extensor strength and BPAD for MBA performance. MBA was selected as the variable of interest for this level of analysis as it represents a composition of multiple aspects of functional mobility. Chronological age and sex were included in regression Model 1 to isolate the effects of the covariates. Model 2 included lower extremity isokinetic extensor strength and BPAD in addition to the covariates in Model 1. Independent contributions of each predictor variable were calculated using the semi-partial r^2^ (_*sp*_r^2^) and the variance inflation factor (VIF) was used to evaluate collinearity of the predictors.

Lastly, to understand whether the relationship between strength and mobility depends on BPAD (i.e., presence of an interaction effect between isokinetic leg extensor strength and BPAD), a moderation analysis was conducted for isokinetic leg extensor strength and the MBA. To explore the impact of task difficulty with respect to our dataset, we performed additional moderation analyses on two subcomponents of the MBA, the four-square step test and gait speed during the six-minute walk test. Chronological age and sex were used as covariates for all analyses. Moderation effects were estimated using the PROCESS macro for SPSS as described by Hayes and Matthes (2009) and Hayes (2018), using 5,000 bootstrap samples with 95% confidence intervals. Following the moderation analysis the Johnson–Neyman technique was used to probe for the interaction and to identify ranges of values of the moderator for which the interaction effect is significant (Johnson and Neyman, 1936; Hayes and Matthes, 2009). Due to the exploratory nature of the analyses, correction for multiple comparisons was not performed for any correlations.

## Results

**FIGURE 2:**
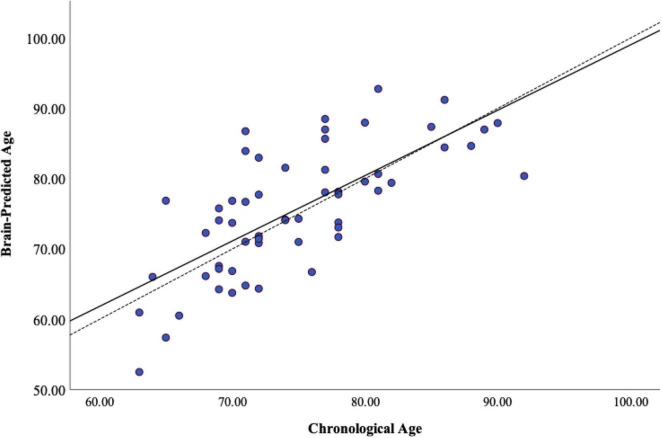
Heterogeneity of brain-predicted age. Brain-predicted age from brainageR regression model. Scatterplot depicting chronological age (x-axis) by brain-predicted age (y-axis). Dashed line is the line of identity and solid black line is the regression line of chronological age on brain-predicted age.

Participants were only included in the present analysis if they had complete data for all variables of interest. Three additional participants were excluded due to poor T1 image quality (e.g., excessive artifact), resulting in data from fifty-seven participants (68% women) available for statistical analyses. Scanner reliability was considered good-excellent with an ICC = 0.973 (CI 0.699–0.996). Statistical assumptions for correlation and linear regression analyses were satisfied. Detailed demographic information and average performance on functional measures are provided in Table 2. In our sample, average BPAD was positive, reflecting advanced brain aging and as expected, there was a significant amount of heterogeneity in BPAD between study participants (Figure 2). There were no significant differences in BPAD with respect to sex (*p* = 0.504).

**TABLE 2:** Participant demographics and functional performance.

Chronological age (yrs)	74.70 ± 6.93
Brain-predicted age difference (yrs)	0.801 ± 6.29
Height (cm)	164.07 ± 10.26
Weight (kg)	72.06 ± 15.66
Body mass index (kg/cm^2^)	26.71 ± 5.14
Body fat (%)	35.04 ± 8.09
Appendicular lean mass/height^2^ (kg/cm^2^)	6.70 ± 1.19
Relative leg extensor strength (N-m/kg body weight)	86.16 ± 32.54
Accelerometry min/wk of moderate–vigorous activity	84.41 ± 56.23
Overall mobility battery assessment score	0.045 ± 0.972
*6-minute walk test (m/sec)*	1.34 ± 0.31
*Four square step test (sec)*	9.80 ± 3.87
*Stair climb power (watts)*	291.49 ± 106.51
*5× chair rise (sec)*	10.85 ± 3.62
*Complex functional task (sec)*	10.01 ± 8.65

*yrs, years; cm, centimeters; kg, kilograms; N, Newtons; m, meters; wk, week; sec, seconds.*

**FIGURE 3:**
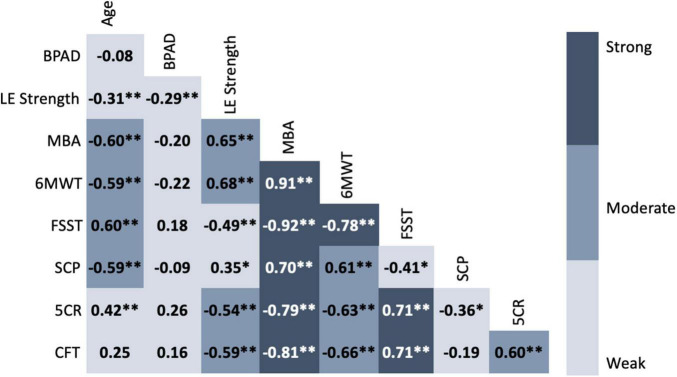
Correlation matrix of chronological age, brain-predicted age difference, normalized leg strength and functional performance. Values represent Pearson’s *r* for each bivariate correlation. *Weak* = 0.00–0.49, *Moderate* = 0.50–0.69, *Strong* = 0.70–1.0 (Jurs et al., 1998). BPAD, brain-predicted age difference; LE, lower extremity; MBA, mobility battery assessment; 6MWT, six-minute walk test; FSST, four square step test; SCP, stair climb power; 5CR, five times chair rise; CFT, complex functional task.

Bivariate correlations revealed moderate to strong relationships between MBA composite, most functional subtests, normalized leg extensor strength and chronological age. In contrast, BPAD was weakly correlated with the other variables of interest (Figure 3). When controlling for both chronological age and sex, isokinetic leg extensor strength normalized to body weight was significantly correlated with BPAD, composite MBA score, 6-min walk test gait speed, four square step test, 5× chair rise time, and time to complete a complex functional task The addition of BPAD as a covariate appeared to weaken the relationship between isokinetic leg extensor strength, composite MBA score, 6-min walk test gait speed, 5×-chair rise time, four square step test and time to complete a complex functional task. However, *r* to Z transformation analyses did not reveal any significant correlational differences when adding BPAD as a covariate. Isokinetic leg extensor strength was not significantly related to stair climb power in either scenario (Table 3).

**TABLE 3:** Correlations between lower extremity strength and functional performance measures.

Covariates	Chronological age	Chronological age	
	Sex	Sex	
		BPAD	
BPAD	−0.317*	N/A	*Z* Value
Overall MBA Score	0.541**	0.493**	0.34 (*p* = 0.734)
*6MWT*	0.585**	0.537**	0.36 (*p* = 0.72)
*FSST*	−0.367*	−0.306*	−0.36 (*p* = 0.72)
*SCP*	0.130	0.122	N/A
*5CR*	−0.491**	−0.434**	−0.38 (*p* = 0.70)
*CFT*	−0.489**	−0.467**	−0.15 (*p* = 0.88)

BPAD, brain-predicted age difference; MBA, mobility battery assessment; 6MWT, six-minute walk test; FSST, four square step test; SCP, stair climb power; 5CR, five times chair rise; CFT, complex functional task. *Statistical significance (p < 0.05). **Statistical significance (p < 0.001).

Brain predicted age difference was also significantly correlated with overall MBA score, four square step test, and 5× chair rise time when controlling for both chronological age and sex. However, after including normalized leg extensor strength as a covariate, BPAD was no longer significantly correlated with any functional measure (Table 4).

**TABLE 4:** Correlations between brain-predicted age difference and functional performance measures.

Covariates	Chronological age	Chronological age
	Sex	Sex
		Average leg extensor strength
Average leg extensor strength	−0.317*	N/A
Overall MBA Score	−0.302*	–0.163
*6MWT*	–0.326	–0.183
*FSST*	0.278*	0.183
*SCP*	–0.046	–0.005
*5CR*	0.316*	0.159
*CFT*	0.162	0.009

MBA, mobility battery assessment; 6MWT, six-minute walk test; FSST, four square step test; SCP, stair climb power; 5CR, five times chair rise; CFT, complex functional task. *Statistical significance (p < 0.05). **Statistical significance (p < 0.001).

Both Model 1 (chronological age and sex covariates) and Model 2 (covariates with normalized lower extremity extensor strength and BPAD) were found to be statistically significant predictors of composite MBA score. Model 1 accounted for 46% of the variance of the MBA score (*p* < 0.001), and both chronological age and sex were statistically significant independent predictors, accounting for a unique contribution in the explained variance in Model 1 of 61% (*p* = 0.003) and 31% (*p* < 0.001), respectively. Model 2 explained 63% of the total variance of composite MBA score (*p* < 0.001), but only normalized leg extensor strength and chronological age were significantly individually predictive of composite MBA score with normalized leg strength explaining 35% (*p* < 0.001) and chronological age 44% (*p* < 0.001) of the variability in composite MBA performance. Variance inflation factor values for both models are within a range that is not concerning for collinearity as all values are less than 2 (Hair et al., 2014). Details of the regression analyses are outlined in Table 5.

**TABLE 5:** Regression model summary for the mobility battery assessment (MBA) score.

	Model characteristics		
	s-p r^2^	VIF	*p*-value
**Model 1**			
*R*^2^ = 0.46; *p*-value ≤ 0.001*			
Sex	–0.307	1.001	0.003*
Age	–0.611	1.001	< 0.001*
**Model 2**			
*R*^2^ = 0.63; *p*-value ≤ 0.001*			
Sex	–0.118	1.215	0.168
Age	–0.449	1.173	< 0.001*
Isokinetic strength/BW	0.345	1.484	< 0.001*
BPAD	–0.101	1.128	0.238

*BW, Body weight.*

**FIGURE 4:**
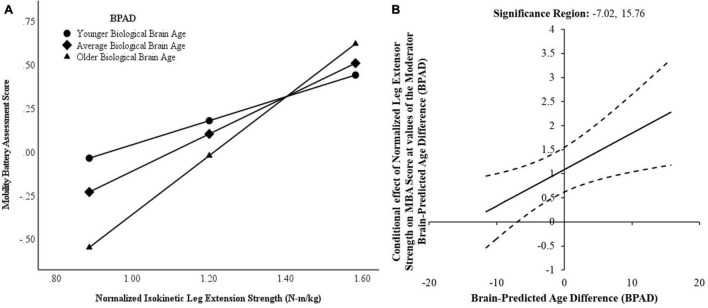
Conditional effects of brain-predicted age difference (BPAD) on the strength-function relationship. **(A)** Normalized leg extensor strength and composite mobility battery assessment (MBA) score demonstrate a weaker relationship for low (younger) brain age (16th percentile). In contrast, normalized leg extensor strength is a stronger predictor of MBA score for average and high (older) brain age (84th percentile). **(B)** Johnson–Neyman plot indicating conditional effects of brain-predicted age difference (BPAD) on the relationship between leg extensor strength and mobility battery assessment (MBA) score performance with a 95% confidence interval (dashed line). Note the vertical boundary lines indicate the range of BPAD where normalized leg extensor strength is a significant predictor of MBA score.

Brain predicted age difference was a significant moderator of the relationship between normalized leg extensor strength and composite MBA score and this interaction accounted for 7.0% of the variance in MBA score [△*R*^2^ = 0.044. *F*(1,51) = 6.83, *p* = 0.01]. Conditionally, normalized leg extensor strength was significantly predictive of composite MBA score when considering mean BPAD and BPAD at the 16th and 84th percentiles. However, the strength of the relationship between normalized leg extensor strength and MBA score varied according to brain age—biologically younger brains (i.e., 16th percentile) attenuated the relationship whereas older brains (i.e., 84th percentile) strengthened the relationship. In addition, the Johnson–Neyman technique revealed a conditional effect of BPAD for very young biological brains (Figure 4). While the relationship between normalized leg extensor strength and MBA was significant within the range of −7.02 to 15.76 years of BPAD, strength was not a significant predictor of functional performance in a small subset of very young brains (i.e., BPAD < −7.02).

Interestingly, BPAD was also a significant moderator of the relationship between normalized leg extensor strength and FSST time [△*R*^2^ = 0.0808. *F*(1,51) = 9.45, *p* = 0.003], but was not a moderator of the relationship between normalized leg extensor strength and gait speed [△*R*^2^ = 0.0061. *F*(1,51) = 0.900, *p* = 0.347]. Conditional moderation effects were also observed with respect to the four-square step test. Specifically, normalized leg extensor strength was only a significant predictor for “average” age brains (i.e., 50th percentile BPAD = −0.32) and “older” brains (i.e., 84th percentile BPAD = 7.8624) but not for four square step test performance in those with relatively “younger” brains (i.e., 14th percentile BPAD = −5.3188).

## Discussion

The primary purpose of this study was to examine the relationship between leg extensor strength and mobility with BPAD as a potential moderator of this relationship in a community-dwelling older population. Our findings indicated that normalized leg extensor strength and BPAD were significantly correlated, and BPAD weakened the association between normalized leg extensor strength and mobility. Interestingly, BPAD was also a significant moderator of the predictive value of normalized leg extensor strength for composite MBA score with an observed conditional effect.

Our current findings replicate the results of prior work, identifying muscle strength as a strong predictor of mobility (Visser et al., 2000; Newman et al., 2006a; Hicks et al., 2012). For example, Davis et al. (1998) reported a positive association between isometric quadriceps strength and functional mobility measures including the five times chair rise test and usual and fast walking speed in a sample of 705 women of Japanese ancestry between the ages of 55 and 93 years old. Similarly, Hairi et al. (2010) examined the relationship between appendicular measures of strength, low lean muscle mass and muscle quality and subjective and objective measures of mobility in a group of 1,705 men older than 75 years of age. Of the predictor variables, muscle strength demonstrated the strongest association with functional mobility (Hairi et al., 2010). Leg strength has also been identified as a potential preclinical marker of mobility decline in older adults. Manini et al. (2007) assessed isokinetic leg extensor strength and functional mobility in the Health, Aging and Body Composition cohort, identifying sex-specific low and high cut-points predictive of future development of severe mobility limitations. For our cohort, both men (1.45 ± 0.49 Nm/kg) and women (1.11 ± 0.33 Nm/kg), on average, demonstrated moderate risk for future mobility limitation based on these cut-points.

Despite the association between muscle strength and mobility, an individual’s ability to generate maximal voluntary force is far from the sole determinant of physical function, leading researchers to call for investigation of additional mechanisms of mobility (Sorond et al., 2015). DiSalvio et al. (2020) identified a relationship between gray matter volume in vestibular, somatosensory and perceptual regions and functional performance as measured by gait speed, balance, and the four square step test, with higher cortical volumes associated with better physical performance. Lu et al. (2020) in a prospective study also reported an association with global gray matter volume and development of frailty—individuals with reduced gray matter volume were more likely to develop frailty than those with higher volumes. In addition, falling as a consequence of impaired mobility has been linked to decreased gray matter, subcortical and lower white matter volume (Hsu et al., 2016).

Given that brain aging encompasses more than one singular aspect of structural integrity (i.e., cortical thickness, white matter volume, etc.), we contend that BPAD may be considered a singular aggregate measure for global brain health and structural integrity to evaluate relative to functional mobility. In line with this approach, Cole et al. (2018) identified a relationship between BPAD and multiple domains of function as well as early mortality. Specifically, a greater BPAD (i.e., relatively “older” brains) was significantly associated with decreased fluid cognition, poorer lung function, weaker grip strength and slower walking speed (Cole et al., 2018). Similarly, our results also suggest a neural influence on function—BPAD was significantly correlated with MBA score and some of the functional subcomponents. However, this relationship was no longer significant when covarying for normalized leg extensor strength, suggesting a more nuanced relationship between BPAD, strength, and function. Indeed, our findings do suggest a more complex interplay between BPAD and muscle strength whereby an interaction between brain age and normalized lower extremity strength resulted in a change in the strength of the relationship between normalized leg extensor strength and MBA composite scores.

Our results also indicate that BPAD has a conditional effect on the predictive value of leg strength for mobility. For individuals with very young brains relative to their chronological age, leg strength was not related to mobility performance. We interpret these findings to suggest that neural processes such as cognition and motor planning may minimize the importance of leg strength for functional performance in young brains. Dual task paradigms are often used to investigate the role of attentional and executive function processes in functional mobility and highlight performance differences in younger versus older adults. Differences in dual task performance across the lifespan may reflect decreased structural integrity of the dorsolateral prefrontal cortex and the orbitofrontal cortex, cognitive brain regions that are particularly sensitive to age-related changes (Raz et al., 2005). For example, in a study comparing young, middle and older aged adults, dual task cost on motor performance in the 10-meter walk test, the Timed Up and Go and the Four Square Step Test increased with age and older adults demonstrated the worst cognitive and motor performance (Brustio et al., 2017).

Anticipatory motor planning also demonstrates age-related decline as measured by end-state comfort effect paradigms. For motor tasks, adults have been shown to plan motor actions in such a manner that initial movement phases result in awkward postures to ensure that later movements lead to a comfortable end posture (Stöckel et al., 2017). However, this anticipatory planning capacity declines with age beginning in the seventh decade of life. In a bar-transport-task, older adults exhibited decreased end-state comfort sensitivity compared to younger adults, an observation exacerbated with increasing task complexity. While the mechanisms underlying this behavior are not entirely clear, older adults’ inability to plan motor actions to increase end state comfort is theorized to reflect a decline in executive and/or motor function. Collectively, age-related decline in dual task and end state effect motor tasks suggests that in younger brains, neural processes (i.e., cognition, motor planning, etc.) may be the preferred mechanism of adequate motor performance.

Alternatively, leg strength may be a compensatory mechanism to maintain physical functioning in the context of advancing brain age. This interpretation of compensation to maintain function is consistent with theories of aging and cognition. For example, the Compensation-Related Utilization of Neural Circuits Hypothesis (CRUNCH) posits that frontal region overactivation in older compared to younger adults is compensatory for age-related changes (i.e., decreased gray matter volume, compromised sensory input) and serves to maintain cognitive task performance (Reuter-Lorenz and Cappell, 2008). CRUNCH also suggests with increasing task difficulty, frontal overactivation reaches a maximal “crunch point,” resulting in insufficient compensatory reserves and impaired performance. The Scaffolding Theory of Aging and Cognition-Revised (STAC-r) takes a broader view of aging, compensation and cognitive performance (Reuter-Lorenz and Park, 2014). Specifically, aging is viewed as a neural insult resulting in structural and functional connectivity changes that necessitate the utilization of scaffolds to maintain cognitive function. In this context, scaffolds can represent variable compensatory mechanisms including increased frontal recruitment (Reuter-Lorenz and Cappell, 2008), recruitment of homologous contralateral regions (Cabeza, 2002) and neurogenesis (Fuchs and Flügge, 2014). Our data demonstrate a similar compensatory pathway for physical function and mobility, whereby increased strength can sustain mobility despite age-related neural structural decline.

Evidence highlighting a “common cause” of neural decline in cognitive, sensory and motor systems (Christensen et al., 2001) as well as observed overactivity in frontal and parietal regions in older individuals during motor tasks (Mattay et al., 2002) supports the adaptation of these theories to our current findings. Thus, we suggest greater BPAD derived from structural neuroimaging inherently reflects accelerated decline in structural brain integrity, resulting in the need for compensatory mechanisms to maintain physical function. At some point in brain aging, neural compensatory mechanisms likely reach maximum utility (i.e., crunch point). Based on our data we postulate that the crunch point of central mechanisms results in the need for additional *peripheral* scaffolding, namely, leg extensor strength, to maintain MBA performance. While not observed on our sample, a peripheral crunch point could theoretically also be reached whereby strength can no longer compensate for advancing neural insufficiency, resulting in a decline in functional mobility. Alternatively, individuals with muscle weakness may lack additional peripheral scaffolding, accelerating the loss of physical function in the context of neural decline.

These views are supported in part by observations in individuals with Alzheimer’s and Parkinson’s disease, two forms of central nervous system pathology shown to have advanced brain-predicted age (Franke et al., 2010; Beheshti et al., 2020). Recently, Sampaio et al. (2020) reported an interesting relationship between lower extremity strength, cognition and functional capacity. In their sample of institutionalized adults with dementia, lower extremity strength was not associated with cognition but was significantly associated with level of independence in activities of daily living (Sampaio et al., 2020). Similarly, progressive resistance and functional training in a cohort of 62 individuals with dementia resulted in significant improvements in both lower extremity strength and performance on a modified Short Physical Performance Battery and cognitive status was not a predictor of the training response (Hauer et al., 2012). In a group of 40 individuals with mild-moderate Parkinson’s disease, low muscle power was significantly associated with walking speed even when controlling for Unified Parkinson’s Disease Rating Scale motor scores (Allen et al., 2010).

**FIGURE 5:**
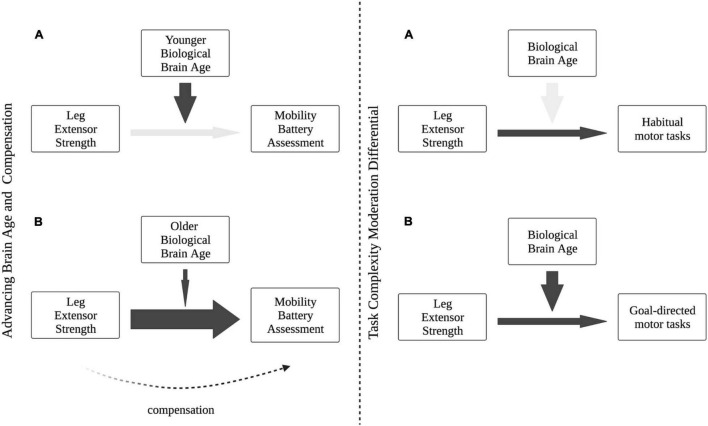
Conceptual framework of our proposed Motoric Aging and Compensation Hypothesis (MACH). **(Left panel A)** In the context of relatively “younger” brains and adequate neural mechanisms of mobility, strength is not needed as a functional compensation. **(Left panel B)** With accelerated brain age and decline in neural integrity, strength becomes a stronger predictor of functional performance and maintains functional capacity. **(Right panel A)** Brain age difference does not moderate the predictive relationship between strength and habitual motor tasks (e.g., gait), indicating neural processing may not be as integral during simpler, more automatic mobility tasks. **(Right panel B)** Increased task complexity of goal-directed motor tasks necessitates greater neural contribution to functional performance, with brain age moderating the relationship between strength and mobility.

Theories of cognitive aging also consider the influence of task difficulty in compensatory activation of alternative neural scaffolds during cognitive tasks. According to CRUNCH, increased neural recruitment in response to increased task complexity is normal—both younger and older adults demonstrate frontal overactivation in response to increase task difficulty to maintain cognitive performance. However, older adults utilize compensatory resources earlier than younger counterparts for the same task. Our results suggest a similar influence of task difficulty on compensation in motor function—BPAD was not a significant moderator of the relationship between leg extensor strength and gait speed, a task that represents a less complex form of functional mobility (i.e., a “habitual” or “automatic” motor task). However, BPAD was a significant moderator when considering the relationship between leg extensor strength and the four-square step test, a task that incorporates additional elements of mobility complexity including change of direction and increased postural stability demands (i.e., a “goal-directed” task). While the results of these exploratory analyses should be interpreted with caution, they may reflect a nuanced compensatory task differential with regard to the relationship between BPAD and leg strength. While more work is certainly needed to identify specific mechanisms of neural and muscular motor compensation with age, a conceptual framework for brain aging, mobility performance and task complexity is presented in Figure 5.

There are several limitations inherent to our study that should be recognized. First, our sample size was relatively small and predominantly female, limiting the generalizability of our findings. Additionally, all subjects in this study were community-dwelling older individuals and as such these results may not be generalizable to all older adults. The cross-sectional nature of this study design also prevents any interpretation of causality or application of these findings to changes over time. Finally, the specific machine learning algorithm used in this study prevents any definitive interpretation of which structural parameters (i.e., gray matter volume, ventricle size, etc.) are most influential in calculating BPA.

## Conclusion

We sought to investigate the moderation effects of brain age on the relationship between leg extensor strength and mobility tasks in older adults. Based on our findings, the previously highlighted relationship between lower extremity strength and physical function appears to be influenced by accelerated brain aging, with lower extremity strength serving as a possible compensation for a decline in neural integrity under more complex conditions. Collectively, these findings underscore the need to explore the nuances of the brain-muscle-function relationship in order to adequately address mobility decline associated with aging.

## Data Availability Statement

The raw data supporting the conclusions of this article will be made available by the authors, without undue reservation.

## Ethics Statement

The studies involving human participants were reviewed and approved by Ohio University Institutional Review Board. The patients/participants provided their written informed consent to participate in this study.

## Author Contributions

BV, JS, DG, and BC: conception and design of the study and drafting a significant portion of the manuscript or figures. All authors: acquisition and analysis of data.

## Conflict of Interest

In the past 5-years, BC has received research funding from NMD Pharma, Regeneron Pharmaceuticals, Astellas Pharma Global Development, Inc., and RTI Health Solutions for contracted studies that involved aging and neuromuscular related research. In the past 5-years, BC has received consulting fees from Regeneron Pharmaceuticals, Zev industries, and the Gerson Lehrman Group for consultation specific to age-related neuromuscular weakness. BC is a co-founder with equity of OsteoDx Inc. The remaining authors declare that the research was conducted in the absence of any commercial or financial relationships that could be construed as a potential conflict of interest.

## Publisher’s Note

All claims expressed in this article are solely those of the authors and do not necessarily represent those of their affiliated organizations, or those of the publisher, the editors and the reviewers. Any product that may be evaluated in this article, or claim that may be made by its manufacturer, is not guaranteed or endorsed by the publisher.

## Footnotes

1
https://github.com/james-cole/brainageR
